# Interoceptive Attentiveness Induces Significantly More PFC Activation during a Synchronized Linguistic Task Compared to a Motor Task as Revealed by Functional Near-Infrared Spectroscopy

**DOI:** 10.3390/brainsci12030301

**Published:** 2022-02-23

**Authors:** Michela Balconi, Laura Angioletti

**Affiliations:** 1 International Research Center for Cognitive Applied Neuroscience (IrcCAN), Università Cattolica del Sacro Cuore, 20123 Milan, Italy; michela.balconi@unicatt.it; 2 Research Unit in Affective and Social Neuroscience, Department of Psychology, Università Cattolica del Sacro Cuore, 20123 Milan, Italy

**Keywords:** interoceptive attentiveness, fNIRS, PFC, synchronization, cognitive tasks

## Abstract

Currently, there is little understanding of how interoceptive attentiveness (IA) affects brain responses during synchronized cognitive or motor tasks. This pilot study explored the effect of explicit IA manipulation on hemodynamic correlates of simple cognitive tasks implying linguistic or motor synchronization. Eighteen healthy participants completed two linguistic and motor synchronization tasks during explicit IA and control conditions while oxygenated (O2Hb) and deoxygenated (HHb) hemoglobin variations were recorded by functional Near-Infrared Spectroscopy (fNIRS). The findings suggested that the brain regions associated with sustained attention, such as the right prefrontal cortex (PFC), were more involved when an explicit focus on the breath was induced during the cognitive linguistic task requiring synchronization with a partner, as indicated by increased O2Hb. Interestingly, this effect was not significant for the motor task. In conclusion, for the first time, this pilot research found increased activity in neuroanatomical regions that promote sustained attention, attention reorientation, and synchronization when a joint task is carried out and the person is focusing on their physiological body reactions. Moreover, the results suggested that the benefits of conscious concentration on physiological interoceptive correlates while executing a task demanding synchronization, particularly verbal alignment, may be related to the right PFC.

## 1. Introduction

Within the dimensions of interoception, interoceptive attentiveness (IA) has been generally described as a top-down process consisting of “the focused attention to a particular interoceptive signal for a specified time interval” [1,2]. IA was formerly conceived as a higher-level dimension that could be regulated and developed through mindfulness-based interventions, controlled breathing, or even short relaxation treatments [3,4]. Interestingly, the consciously focused attention on breath that underpins these practices has been shown to improve several cognitive and emotional processes including sustained attention, cognitive monitoring, and meta-awareness (as examples of cognitive processes focused on interoceptive inputs [4]), the regulation of emotions (such as the reduction in negative affect [5], and a higher level of receptivity and less oversensitivity to internal sensory stimuli) [6,7], the empathic resonance of empathy for pain [8], and stress management (understood as the ability to regain concentration even in the most demanding conditions) [9]. However, to date little is known about how deliberate attention to interoceptive correlates affects performance during a social interaction that requires or necessitates synchronization with the other partner of the dyad, such as a communication process, a teamwork dynamic, or a general interpersonal relationship.

Starting from the neural bases that support IA, previous neuroscientific research has demonstrated activation of different portions of the right hemisphere, such as the supramarginal (SM) gyrus [10,11,12,13,14], the prefrontal cortex (PFC) [15], the dorsolateral prefrontal cortex (DLPFC) [16], and the frontopolar cortex [17] during the execution of interoceptive attention/awareness (IAA) tasks. While the right SM has been shown to be the “neural coder” of peripersonal space and corporeal awareness [10], the right DLPFC, instead, seems to orient and sustain attention by avoiding distractions in a goal-directed way [18]. It also helps to better maintain focus on the breath, by increasing the awareness of the person when their mind wanders and thus shifts the attention back to the breath [12]. Additionally, the involvement of the right frontopolar cortex was demonstrated in the evaluation of alternative courses of action [19], resource allocation [20], and direct exploration [21]. Finally, the DLPFC has also been associated with social processes including cooperation among opposite-sex partners [22], social cognition [23], reciprocal cooperative interactions and interpersonal coordination [24,25].

With reference to the studies that adopt synchronization tasks in the neuroscientific literature, synchronization is most frequently studied with movement or language behavioral synchrony [26]. Studies show that synchronous movement and speech increases affiliation [27], success in cooperative tasks [28], compassion and inner group altruism [29], and prosocial behavior towards out-group members [30].

Previous research explored the neural correlates of synchronization during simple motor and linguistic instructed imitation tasks. In particular, the development of the hyperscanning paradigm [26,31] revealed inter-brain coupling as a novel discovery for understanding the neurological mechanisms driving interpersonal synchronization. For instance, during joint-tapping tasks, the prefrontal regions (particularly the right PFC) of two interacting agents showed synchronization [22,32,33,34,35,36]. This paradigm has also been used to explore live interactive speech, effectively expanding neuroscientific accounts of live verbal communication and social engagement [37,38,39,40,41,42]. In a recent systematic review of hyperscanning studies on spoken communication and language [43], it was stated that neural synchronization predominantly involved the frontal and temporo-parietal regions, which support the mirroring and mentalizing mechanisms that are ongoing during communication processes. Despite the predominant role of the left hemisphere in language processing and face-to-face verbal conversations [37], there is evidence of right-sided activations of frontal (right DLPFC) and temporal regions (right temporo-parietal junction, rTPJ) during naturalistic paradigms involving verbal cooperation and turn-taking [40,44].

However, the current literature on the neural correlates of IA and on the impact of IA on synchronized tasks is still scarce and has not been studied with classical neuroimaging methods, such as functional Magnetic Resonance Imaging (fMRI). Because it gives the most precise estimate of functional activity in the entire brain, fMRI is considered a reference method in neuroimaging research. During fMRI experiments, participants must stay completely motionless, and even the tiniest movement might cause data mistakes or exclusion [45]. Accordingly, because of its sensitivity to movement artifacts, fMRI cannot be employed during real social interactions or movements, while functional Near-Infrared Spectroscopy (fNIRS) can be a useful technique that can overcome these limitations [46]. Indeed, the paucity of fMRI studies on IA tasks or brief relaxation practices could be due to the difficulty of an individual executing these practices in the fMRI scanner [17], and this proves even more difficult to perform during motor synchronization tasks. In contrast, fNIRS is particularly well-suited for evaluating the cortical responsivity in terms of hemodynamic variations related to information processing even in the presence of movement artifacts [47,48]. Despite the quite low spatial resolution and the lack of coverage of the subcortical areas if compared to fMRI, the fNIRS system has a low sensitivity to body movements, it is portable, easy to use, non-invasive, and makes it possible for participants to perform synchronized tasks in a more naturalistic way [49]. In addition, previous neuroscientific research adopted fNIRS to explore the cortical correlates of brief relaxation practice during arithmetic tasks [17], and the hemodynamic responses associated with the manipulation of IA in healthy individuals during observation of pain stimuli [16]; however, these studies did not include cognitive synchronization tasks.

The impact of IA manipulation on human social interactions is an important research topic for promoting self-regulation during interpersonal relations, for a better communication process, for empathic responses, and even real-life social interaction dynamics, such as teamwork. Indeed, we are interested in investigating the role of IA manipulation, as a potential interoceptive intervention, in creating synergy between individuals, in particular by enhancing its effectiveness in conditions of interaction (such as during a communicative exchange). This also benefits self-regulation since being able to accurately “tune in” to one’s own internal (emotional) states and properly use them in social interactions may improve social connection [50].

Hence, this pilot study aims to test the effect of explicit IA manipulation on hemodynamic correlates of simple tasks that involve linguistic or motor synchronization. It is supposed that the brain regions associated with sustained attention, such as the right DLPFC area, will be more involved when inducing an explicit IA focus on the breath during a cognitive task requiring synchronization with a partner. According to the current knowledge, this work focused on the PFC regions as the most significant neuroanatomical structures that support attentional processes during cognitive tasks [51] and play a key role in social cognitive interactions requiring synchronization [22,52,53].

Based on the literature and evidence presented above, we hypothesized that firstly, IA could expand the effects of increasing the brain response to synchronization for cognitive tasks, thus more so for linguistic than motor tasks, in line with previous evidence supporting the positive impact of IA (in terms of self-regulation) on cognitive processes [4]. The focus on oneself could augment the capability to read the signals and the feedback of the interaction with the other member of the dyad. Secondly, the specific PFC activation, which is deputed to support interpersonal synchronization [22,25], could be increased (in terms of increased O2Hb) by the explicit IA condition, which also requires the engagement of more attentive resources. Thirdly, according to the literature [10,18,35], there could be a possible lateralization effect, for which the right PFC, which is highly involved in goal-directed attention, could be more activated than the left one in the explicit IA condition during the cognitive synchronization tasks.

## 2. Materials and Methods

### 2.1. Sample

A total of eighteen healthy participants (14 females and 4 males; mean age (*M*) = 27.05; standard deviation (*SD*) = 3.18) were recruited by word of mouth for this fNIRS pilot study. A convenience non-probabilistic sampling process was adopted by involving university students. Given that the examined phenomenon is relatively novel in the field of social neuroscience and the literature did not provide systematic repeated evidence, it was not possible to exploit former references to estimate the size of the expected significant effects. Therefore, in order to estimate a minimum needed sample size, we ran a priori power analysis for repeated measures ANOVA and a total sample size (with alpha error probability = 0.5 and power 0.80) of 17 was the minimum for detection of a significant within effect or interaction between factors (G*Power 3.1 software, Heinrich-Heine, Germany [54]).

Physiologic conditions of chronic or acute pain, significant medical and chronic diseases, seizures, traumatic brain injury, pregnancy, previous meditation experience, and any mental or neurologic issue were all considered exclusion criteria. Included participants had normal-to-corrected vision and were right-handed. Before the experiment, all participants signed a written informed consent form. They received no remuneration for their participation in this pilot study. Approval for the study was provided by the Department of Psychology of the Catholic University of the Sacred Heart of Milan, Italy, and it was conducted in accordance with the Declaration of Helsinki.

### 2.2. Experimental Procedure

The experimental phase was performed in a dimly lit room, and participants were seated in front of an experimenter who provided the experimental instructions and executed the tasks.

Participants were required to execute simple linguistic and motor synchronization tasks by imitating the experimenter while fNIRS hemodynamic measures were recorded. For the linguistic synchronization task, participants had to syllabize with the experimenter for a total of three minutes (modified version of the human-to-human alternating speech task [55]). In this modified version of the alternating speech task, the participant was asked to pronounce four syllables “LA”, “BA”, “CA”, “DA” sequentially and alternately; for instance, the experimenter said “LA” and then the participant said “LA” and so on. The rhythms of the speech were not established a priori. Each linguistic synchronization task session lasted three minutes, without intervals. The average number of loops (that is the number of times from “LA” to “DA”) was no less than 45 for the three minutes.

The motor synchronization task consisted of a simple finger movement task (modified version of the task adopted in a previous study [56]) in which participants had to synchronize their finger movements with the experimenter sitting in front of them for a total duration of three minutes. Specifically, in the finger movement task, the participants were instructed to place their hands on the table in the prone position, with the fingers about one centimetre apart and elbows on the table. They next elevate their dominant hand’s fingers and tap the table with their little, ring, middle, index finger, and thumb. They were not instructed to execute this movement at a specific speed or to raise their fingers as high as possible, they were only instructed to synchronize with the movement performed by the experimenter seated in front of them.

The order of task execution was randomized and counterbalanced to prevent potential biases due to sequence effects.

**Figure 1:**
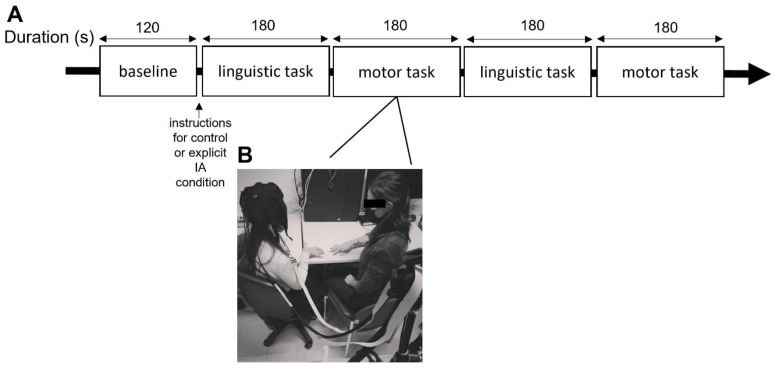
Experiment setup. (**A**) The experimental procedure with the synchronization tasks duration and timing of the instructions provided for the explicit IA and control conditions. (**B**) Example of the experimental setting with fNIRS device recording and the experimenter during the task execution (i.e., for the motor task).

All the participants executed these two tasks in two different conditions: the explicit IA condition and a control condition [8,16]. In the explicit IA condition, the participant was also explicitly required to focus on the interoceptive changes while performing the task and received the following instruction, “During this task, we ask you to focus your attention on your bodily sensations (such as the breath). Try to observe how you feel and if there are any variations in your body as you perform the task”. In the control condition, the participant received the general instruction to perform the tasks without the explicit request to focus their attention on their interoceptive correlates. A resting baseline lasting 120 s was registered at the beginning of the experiment. The whole experimental phase lasted less than 30 min (for a graphical representation of the procedural steps, see Figure 1A,B).

### 2.3. fNIRS Data Acquisition and Biosignal Analysis

A six-channel optodes matrix of an NIRScout System (NIRx Medical Technologies, LLC, Los Angeles, CA, USA) was utilized to record the hemodynamic signal in terms of variations in oxygenated hemoglobin (O2Hb) and deoxygenated hemoglobin (HHb) concentrations. Four light sources/emitters and four detectors were placed over the scalp using a fNIRS cap in accordance with the standard international 10/5 system [57].

**Figure 2:**
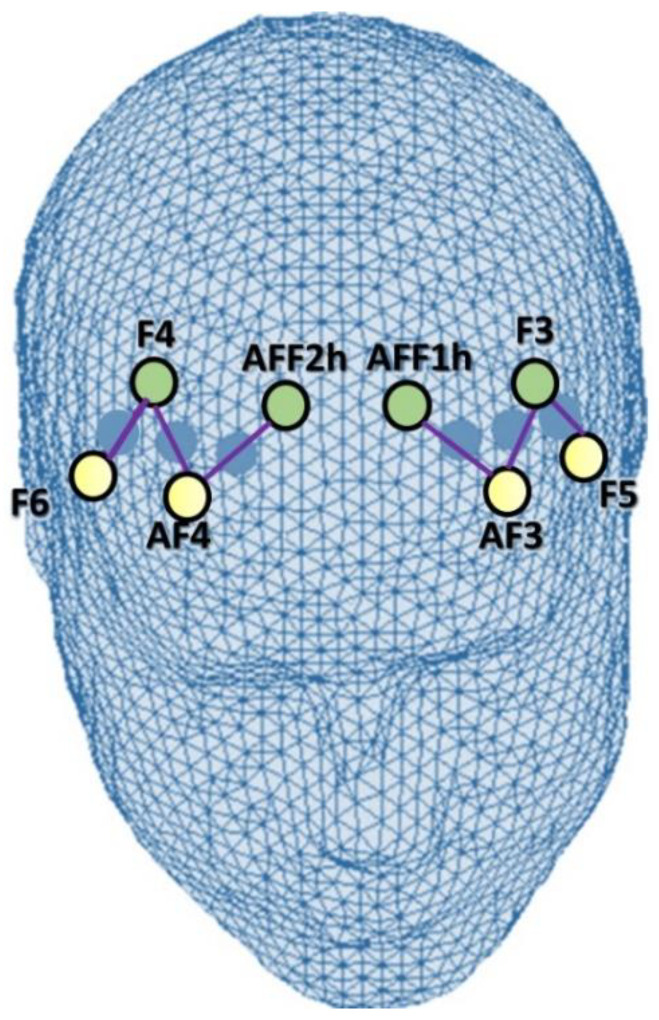
fNIRS montage. Location of the sources (yellow) and detectors (green) of fNIRS montage. Sources were in the following positions: AF3-AF4, F5-F6. Detectors were placed on: AFF1h-AFF2h, F3-F4. A total of six channels (violet) have been acquired: Ch1 (AF3-F3), Ch2 (AF3-AFF1h), Ch3 (F5-F3), Ch4 (AF4-F4), Ch5 (AF4-AFF2h), Ch6 (F6-F4).

The fNIRS montage included four emitters positioned at AF3-AF4, F5-F6, and four detectors located at AFF1h-AFF2h, F3-F4. For contiguous optodes, the emitter–detector distance was preserved at 30 mm, and it employed a near-infrared light with two wavelengths (760 and 850 nm). Using this arrangement of the optodes, it was possible to acquire a total of six channels: Ch1 (AF3-F3), Ch2 (AF3-AFF1h), Ch3 (F5-F3), corresponding to the left PFC, and Ch4 (AF4-F4), Ch5 (AF4-AFF2h), Ch6 (F6-F4) consistent with the right PFC [16,58] (Figure 2). The locations of the sources and detectors, as well as the area between them, were associated with the best underlying functional region and the most suitable Brodmann area. For this purpose, various references and online atlases were consulted [59,60].

The variations in the concentrations of O2Hb and HHb were recorded constantly during the tasks by using NIRStar Acquisition Software (NIRx Medical Technologies LLC, Glen Head, NY, USA) and beginning with a 120 s resting baseline. The responses derived from the six channels were collected at a sample rate of 6.25 Hz, then processed and converted using the nirsLAB software (v2014.05; NIRx Medical Technologies LLC, 15 Cherry Lane, Glen Head, NY, USA), based on their wavelength and position, resulting in mmol × mm values corresponding to the variations in the concentration of O2Hb and HHb per channel. The raw signal of O2Hb and HHb that was collected from each channel has been digitally band-pass filtered at 0.01–0.3 Hz.

**Figure 3:**
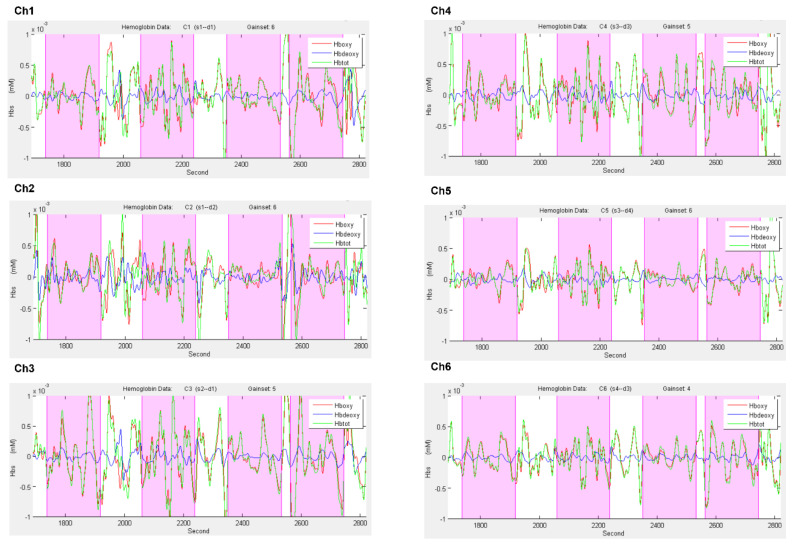
Hemodynamic signal time course for all channels under the four conditions. The figure shows the time course plots of O2Hb (red) and HHb signal (blue) for each channel when performing the following tasks: motor and linguistic tasks during the control condition and motor and linguistic tasks during the explicit IA condition.

To detect noisy channels due to motion artifacts or amplitude changes, raw time-series were visually inspected subject-by-subject both during the experimental phase and the signal analysis. Three percent of the data was eliminated for artifacts. During this visual inspection, channels with poor optical coupling and absence of ~1 Hz heartbeat oscillations were excluded [61]. Moreover, a linear-phase FIR filter on respiration (0.3 Hz), that obtains the symmetric-impulse-response, was used [62,63]. Figure 3 shows the plots of the time course of O2Hb and HHb for all channels under the four conditions.

After the biosignal analysis, the mean concentration of each channel was derived for the tasks. The effect size in each condition was determined based on the mean concentrations in the time series for each channel and subject. The effect sizes (Cohen’s d) were computed by dividing the difference between the baseline and trial means by the baseline standard deviation (SD): D = (m1 − m2)/s, where m1 and m2 are the mean concentration levels for the baseline and trial, respectively, and s is the baseline SD. To optimize the signal-to-noise ratio, the effect sizes from the 6 channels were averaged. Whereas raw fNIRS data were originally relative values that could not be directly averaged across participants or channels, normalized effect size data were averaged regardless of the unit since effect size is unaffected by the differential pathlength factor (DPF).

For the statistical analysis of the fNIRS data, two Regions of Interest (ROI) grouping, the frontal left (Ch1-Ch2-Ch3) and right (Ch4-Ch5-Ch6) homologous channels were used and conceived of as corresponding to the left and right PFC.

### 2.4. Statistical Analysis

A set of repeated measures ANOVAs with independent within factors Condition (2: Explicit IA, Control) × *Task* (2: Motor, Linguistic) × *Lateralization* (2: Left, Right) was applied to D dependent fNIRS data (O2Hb and HHb concentration values). Pairwise comparisons were applied to the data in case of significant effects. Simple effects for significant interactions were further checked via pairwise comparisons, and Bonferroni correction was used to reduce multiple comparisons potential biases. For all the ANOVA tests, the degrees of freedom were corrected using Greenhouse–Geisser epsilon where appropriate. Furthermore, the normality of the data distribution was preliminarily assessed by checking kurtosis and asymmetry indices. The size of statistically significant effects has been estimated by computing partial eta squared (*η*^2^) indices.

## 3. Results

### fNIRS Results Subsection

The following results concern the statistical analyses applied to D dependent measures for O2Hb and HHb concentration values.

**Figure 4:**
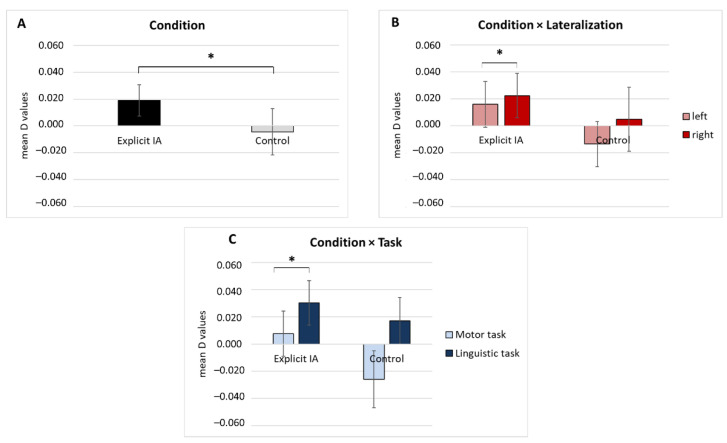
fNIRS hemodynamic results. (**A**) The graph displays O2Hb modulation (D values) as a function of Condition, which is significantly increased for the explicit IA compared to the control condition. (**B**) The bar chart shows significantly higher O2Hb values in the right compared to left frontal areas in the explicit IA condition. (**C**) As the bar graph shows, significantly greater mean O2Hb values were found in the linguistic than the motor task in the explicit IA condition. All data are represented as mean ± SE; all asterisks (*) mark statistically significant differences, with *p* ≤ 0.05.

For the O2Hb signal a first significant main effect was found for the Condition (*F*[1, 17] = 4.56, *p* = 0.02, *η*^2^ = 0.490) (Figure 4A).

Secondly, a significant interaction effect Condition × Lateralization was detected for O2Hb values (*F*[1, 17] = 5.89, *p* = 0.01, *η*^2^ = 0.502). Pairwise comparisons showed significant higher mean values in the right compared to left frontal areas in the explicit IA condition (*F*[1, 17] = 459, *p* = 0.035, *η*^2^ = 0.378) (Figure 4B).

A third significant interaction effect Condition × Task was identified for O2Hb values (*F*[1, 17] = 5.09, *p* = 0.02, *η*^2^ = 0.430). According to pairwise comparisons, significantly higher mean values were found in the linguistic than in the motor task in the explicit IA condition (*F*[1, 17] = 6.09, *p* = 0.01, *η*^2^ = 0.55) (Figure 4C).

No significant effects were found for the HHb values.

## 4. Discussion

The present fNIRS pilot study analyzed the effect of explicit IA manipulation on hemodynamic correlates of simple cognitive tasks implying linguistic or motor synchronization. In line with our hypotheses, we found that brain regions associated with sustained attention, such as the right frontal areas (PFC), were more involved when the explicit focus on the breath was induced during the cognitive task requiring synchronization with a partner. Interestingly, this effect was significant for the linguistic but not for the motor task.

As a first result, it was shown that IA could expand the effects of increasing the brain response to synchronization for cognitive tasks. This evidence was in line with previous studies that support the positive impact of IA and brief relaxation practices on several cognitive functions, such as sustained attention, cognitive monitoring, and meta-awareness [4], but also on compelling arithmetic tasks [17]. In this context, the explicit IA focus could perhaps have expanded these effects by increasing the brain response to synchronization for a simple cognitive task, especially with a significant result for the linguistic and not for the motor task. This result could potentially be explained as the effect of the attention to bodily correlates, which consequently impacts mental functioning (in particular, in the linguistic domain).

Secondly, given the nature of the predominant nature of the task requiring synchronization, the IA effect became even more evident in the linguistic task than in the motor synchronization task. This effect seems to be counterintuitive if we consider the direct relationship between interoceptive correlates (and the attention devoted to control them) and motor performance, and also considering the neuroanatomic proximity between the interoceptive and the motor area; however, this was not the case, and the linguistic task seems to “benefit” more from the IA manipulation. A possible explanation can be found in the nature of the linguistic cognitive task requiring mentalizing abilities, verbal cooperation, and turn-taking. Indeed, in previous hyperscanning studies, right-sided activations of the frontal (right DLPFC) and temporal (right temporo-parietal junction, rTPJ) regions have been observed during naturalistic paradigms involving verbal cooperation and turn-taking [40,44]. Thus, a possible explanation for the discrepancy found in the results between the linguistic and motor tasks, could be that the greater synergy induced by the IA in the linguistic task is linked to the cognitive component, which is predominant in this task, and this could have a greater effect on the PFC. On the other hand, the PFC hemodynamic change in the control condition of the motor task was actually reduced. This evidence strengthens the results related to the linguistic task, highlighting that the motor task, without the IA, did not promote a real “cognitive synergy” that was detectable with the increase in the PFC. Indeed, a possible explanation is that the motor synchronization task only, without the explicit IA manipulation, lowers the engagement of the frontal regions, which are deputed to the real synergy between individuals.

Thirdly, the specific PFC activation derived by the synchronization task was increased by the explicit IA condition. The PFC plays a crucial role in promoting interpersonal synchronization [22,25], and here it could have acted as a “soundboard” or an IA amplifier that enhances the effect of IA on linguistic synchronization. Indeed, the activation of the DLPFC has previously been linked to social processes such as partner cooperation [22], mutual collaborative behaviors, and interpersonal tuning [24,25]. However, more confirmative studies are needed to support the interpretations of the results of this pilot study.

Finally, a lateralization effect, for which the right PFC was more responsive than the left one in the explicit IA condition during the linguistic synchronization task, was observed. According to previous studies, the right frontal portions of the cerebral cortex seem to support the execution of IA tasks [15,16,17,35], and the right DLPFC, in particular, appears to support sustained and goal-directed attention [18]. For the first time, this pilot study suggested the involvement of the right DLPFC in tasks involving interpersonal coordination and a simultaneous explicit focus on its interoceptive correlates.

Notwithstanding the left hemisphere’s dominance in language processing and face-to-face verbal communication [37], it is possible that the social nature of the task (implying a synchronization process) and the explicit focus on interoceptive correlates could have promoted a significantly higher activation of the right PFC compared to the left PFC in the linguistic task [64].

Despite the potential innovativeness of this work, there are a few limitations to take into consideration. First of all, the fNIRS technique was only applied over the PFC and not on the entire brain or in the somatosensory cortical regions or subcortical structures [47]. Although these structures have not been covered due to the specific interest in the PFC contribution and the reduced spatial resolution of fNIRS, it would be interesting to develop future studies that delve into the role of these regions during social interactions and IA manipulation to obtain a complete picture of the networks involved in the process. Further research should also include brain control sites. Secondly, the sample size is relatively small; therefore, to generalize these results it should be increased, and gender must be balanced. Thirdly, in the future, it is necessary to control the voluntary component of the respiratory rate, which could influence the overall results of these studies, and compare the difference between tasks that involve the focus on breathing and cardiac activity. Moreover, this study lacks experimental control on the manipulations (we attempted to measure the participants’ self-perception of synchronization; however, no objective measure of synchronization was provided) and this should be integrated in the future.

Fourthly, for this pilot study, two cognitive synchronization tasks were arbitrarily selected (i.e., the linguistic and motor task); however, further work is needed to evaluate the effect of explicit IA on other synchronizations tasks. Finally, it is necessary to consider other settings and types of tasks in addition to the laboratory one, where more naturalistic and real-life conditions are reproduced in which synchronization is required. Thus, future studies should consider including more realistic conditions such as singing, playing an instrument, or creative problem-solving, and at the methodological level, it could be informative to collect the neural activity of a dyad while performing these tasks (for instance by applying the hyperscanning paradigm).

## 5. Conclusions

To summarize, the current pilot study aimed to investigate differences in frontal cortex activation patterns during simple cognitive synchronization tasks performed in two conditions: one in which participants were explicitly required to focus on their interoceptive correlates, and the control condition in which they were simply instructed to complete the tasks. In conclusion, the data revealed that during a linguistic synchronization task, the explicit IA condition was related to significant right frontal lobe activity. The current research, for the first time, found an increase in activity in neuroanatomical regions, which promotes sustained attention, attention reorientation, and synchronization when a joint task is carried out and the person is focusing on their physiological body reactions. Our findings suggest that the benefits of conscious concentration on physiological interoceptive correlates while executing a task demanding synchronization, particularly verbal alignment, may be related to the right PFC.

## Data Availability

The datasets used and/or analyzed during the current study are available from the corresponding author on reasonable request.
